# Weighted gene co-expression network analysis identifies specific modules and hub genes related to coronary artery disease

**DOI:** 10.1186/s12872-016-0217-3

**Published:** 2016-03-05

**Authors:** Jing Liu, Ling Jing, Xilin Tu

**Affiliations:** Department of Cardiology, Harbin the second hospital, Harbin, Heilongjiang 150056 China; Department of Cardiology, First affiliated hospital of Harbin medical university, Harbin, Heilongjiang 150036 China; Emergency Internal Medicine, First affiliated Hospital of Harbin Medical University, Harbin, Heilongjiang 150036 China; Department of Cardiology, First Clinical College of Harbin Medical University, Harbin, Heilongjiang 150001 China

**Keywords:** Coronary artery disease, Weighted Gene Co-expression Network Analysis, Significant modules, Hub genes

## Abstract

**Background:**

The analysis of the potential molecule targets of coronary artery disease (CAD) is critical for understanding the molecular mechanisms of disease. However, studies of global microarray gene co-expression analysis of CAD still remain limited.

**Methods:**

Microarray data of CAD (GSE23561) were downloaded from Gene Expression Omnibus, including peripheral blood samples from CAD patients (*n* = 6) and controls (*n* = 9). Limma package in R was used to identify the differentially expressed genes (DEGs) between CAD and control samples. Using weighted gene co-expression network analysis (WGCNA) package in R, WGCNA was performed to identify significant modules in the network. Then, functional and pathway enrichment analyses were conducted for genes in the most significant module using DAVID software. Moreover, hub genes in the module were analyzed by isubpathwayminer package in R and GenCLiP 2.0 tool to identify the significant sub-pathways.

**Results:**

Total 3711 DEGs and 21 modules for them were identified in CAD samples. The most significant module was associated with the pathways of hypertrophic cardiomyopathy and membrane related functions. In addition, the top 30 hub genes with high connectivity in the module were selected, and two genes (*G6PD* and *S100A7*) were taken as key molecules via sub-pathway screening and data mining.

**Conclusions:**

A module associated with hypertrophic cardiomyopathy pathway was detected in CAD samples. *G6PD* and *S100A7* were the potential targets in CAD. Our finding might provide novel insight into the underlying molecular mechanism of CAD.

## Background

Coronary artery disease (CAD, also named ischemic heart disease, atherosclerotic cardiovascular disease, atherosclerotic heart disease and coronary heart disease) is one of the most common forms of heart disease that remains a leading cause of morbidity and mortality in the entire world population [1, 2]. In 2010, CAD causes more than 7 million deaths worldwide [3]. CAD has a number of risk factors, including family history, obesity, diabetes, hypertension, high blood lipids, smoking, stress and lack of exercise [4]. Although numerous efforts have been undertaken, it remains a major challenge for scientists to prevent and cure this disease. It is predicted that the disease will be the major and the most common threat to human life by the year 2020 [5, 6]. Therefore, it’s urgent to reveal the mechanisms of CAD and develop novel therapeutic strategies.

The clinical manifestations of CAD are heritable traits, and the knowledge of genome variations carrying risk is helpful in improving diagnosis and treatment of CAD. In the past decades, plenty of genetic changes in CAD have been identified, increasing our understanding to the underlying molecular mechanism of CAD. For example, lipase (*LIPA*) gene is proved to be associated with prevalent cardiovascular risk factors for CAD [7]. The gene encoding vascular endothelial growth factor is suggested to be able to augment myocardial perfusion in CAD patients [8]. On the basis of genetic evidence, interleukin 6 receptor (IL6R) blockade is demonstrated to be a potential therapeutic approach for CAD prevention [9]. Furthermore, Chen et al. report that miR-545-TFEC and miR-585-SPOCK1 are highly correlated with CAD [10].

Gene co-expression network based approaches have been widely used in analyzing microarray data, especially for identifying functional modules [11, 12]. Weighted Gene Co-expression Network Analysis (WGCNA) is one of the most useful gene co-expression network based approaches. Malki et al. use WGCNA to construct the gene co-expression network and identify neuro-oncological ventral antigen 1 (*NOVA1*) and ubiquitin specific peptidase 9, X-linked (*USP9X*) in the most significant module are associated with major depressive disorder and pharmacological treatment response [13]. WGCNA identifies Spleen Tyrosine Kinase (*SYK*) as a candidate oncogene in a subset of small-cell lung cancer [14]. Zhao et al. suggest that dedicator of cyto-kinesis 2 (DOCK2), dedicator of cyto-kinesis 8 (DOCK8) and Fc fragment of IgG, low affinity of IIa, receptor (*FCGR2A*) may represent potential therapeutic targets via WGCNA combined with methylation data analysis [15]. Therefore, WGCNA could be used to analyze microarray data of CAD in this research.

To reveal the potential molecular mechanisms of CAD, we investigated the mRNA expression profile of CAD samples to identify the highly connected hub genes and significant modules. WGCNA was used to construct the co-expression network and identify significant modules in the network. As the modules may correspond to biological pathways, detailed analysis of the modules will allow us to understand the molecular mechanisms. In addition, we also identified the highly connected hub genes in the most significant module.

## Methods

### Microarray data

Microarray data of GSE23561 [16] was downloaded from the National Center For Biotechnology Information (NCBI) Gene Expression Omnibus (GEO, http://www.ncbi.nlm.nih.gov/geo/) database [17, 18], which was based on the platform of GPL10775 Human 50 K Exonic Evidence-Based Oligonucleotide Array. GSE23561 contains 6 peripheral blood samples from CAD patients (mean age = 56, 5 males and 1 female) and 9 peripheral blood samples from control individuals who had never been diagnosed with a chronic illness (mean age = 45.9, 2 males and 7 females). CAD patient was diagnosed through detecting flow-limiting coronary artery stenoses using imaging techniques. All of the CAD patients were also treated for systemic hypertension. Grayson et al. deposited GSE23561, and their research was approved by the Institutional Review Board of Vanderbilt University [16]. In the study of Grayson et al., all subjects gave their written informed consent [16].

### Data preprocessing

After GSE23561 was downloaded, probe identification numbers (IDs) were transformed into gene symbols. For multiple probes corresponding to one gene, their average expression value was taken as the gene expression value. After that, gene expression values were normalized using preprocessCore package (version 1.28.0, http://www.bioconductor.org/ packages/release/bioc/html/preprocessCore.html) [19], and were performed with log_2_ transformation.

### Differentially expressed genes (DEGs) screening

Linear models for microarray data (Limma) is a library used for analyzing gene expression microarray data, especially the use of linear models for the assessment of differential expression and the analysis of designed experiments [20]. Limma package (version 1.22.0, http://www.bioconductor.org/packages/release/bioc/html/limma.html) in R was applied to identify the DEGs between CAD samples and control samples. Genes with |log_2_ fold change (FC)| ≥ 0.5 were selected for subsequent analysis.

### Construction of Weighted Gene Co-expression Network

WGCNA is a widely used systems biology method, which is used to construct a scale-free network from gene expression data [11]. At first, the Pearson’s correlation matrices were calculated for all pairs of genes, the correlation coefficient between gene *m* and gene *n* was defined as Smn = |cor(m,n)|. Then, the Pearson’s correlation matrices were transformed into matrices of connection strengths using a power function a_mn_ = power (S_mn_, β) = |S_mn_|^β^ . This step can emphasize strong correlations and reduce the influences of weak correlations on an exponential scale. Here, the power of β = 17 (Soft.R.sq = 0.8) were chose to make sure we can obtain a scale-free network. These connection strengths were used to calculate topology overlap (TO) [21], which measures the connectivity of a pair of genes. In this study, hierarchical average linkage clustering [22] based on TO was used to identify gene co-expression modules, which could group genes with similar patterns of expression. The WGCNA package in R can be used for performing various functions in weighted correlation network analysis, including constructing network, detecting module, calculating topological properties, simulating data, visualization, and interfacing with external software [23]. Using WGCNA package (version 1.46, http://www.inside-r.org/packages/cran/WGCNA/docs/bicor) in R, the analysis was performed as described previously [11, 24].

After the modules were identified, the *T*-test was used to calculate the significant *p*-value of candidate mRNAs, and the gene significance (GS) was defined as mediated *p*-value of each gene (GS = lgP). Then, the module significance (MS) were defined as the average GS of all the genes involved in the module. In general, the module with the highest MS among all the selected modules will be considered as the one associated with disease. In addition, we also calculated the relevance between the feature vector of modules and phenotypes to identify the most relevant module. Gene Ontology (GO) analysis is applied to reveal functions of gene products from three aspects: biological process (BP), cellular component (CC) and molecular function (MF) [25]. The Kyoto Encyclopedia of Genes and Genomes (KEGG) pathway database aims at describing functions of molecules or genes [26]. Using the Database for Annotation, Visualization, and Integrated Discovery (DAVID) software [27], GO functional and KEGG pathway enrichment analyses for the genes in the most significant module searched by the two methods were performed. The *p*-value < 0.05 was used as the cut-off criterion.

### Sun-pathway analysis of hub genes in the most significant module

Hub genes were highly connected nodes which involved in much more interactions. Hub genes in a module may be more important than other genes in the whole network. Gene connectivity can measure the connection strength of a gene connect to other genes in global network. Therefore, hub genes with higher connectivity in the selected module were extracted.

The occurrence of certain diseases is not always caused by the abnormality of the whole pathway involved in the biological process. It is more likely to be caused by dysfunction of the sub-pathways. For this reason, we focused on the sub-pathways of hub genes to narrow down our research. The iSubpathwayMiner can be applied for graph-based reconstruction and analysis of pathways [28]. Using the iSubpathwayMiner package (version 3.0, http://cran.r-project.org/web/packages/iSubpathwayMiner/) in R, the significant sub-pathways of the hub genes were identified (*p*-value < 0.05). The goal of GenCLiP 2.0 is to serve for free tern-based network construction and functional clustering of genes [29]. Thus, GenCLiP 2.0 tool (http://ci.smu.edu.cn/GenCLip/analysis.php) was used to collect the correlated pathways of hub genes.

## Results

### Data preprocessing and DEGs screening

After data preprocessing, the expression matrices of 24277 genes were obtained from the 15 samples. Under the threshold of |log_2_FC| ≥ 0.5, total 3711 DEGs were selected for subsequent analysis.

### WGCNA analysis and key modules identification

**Fig. 1:**
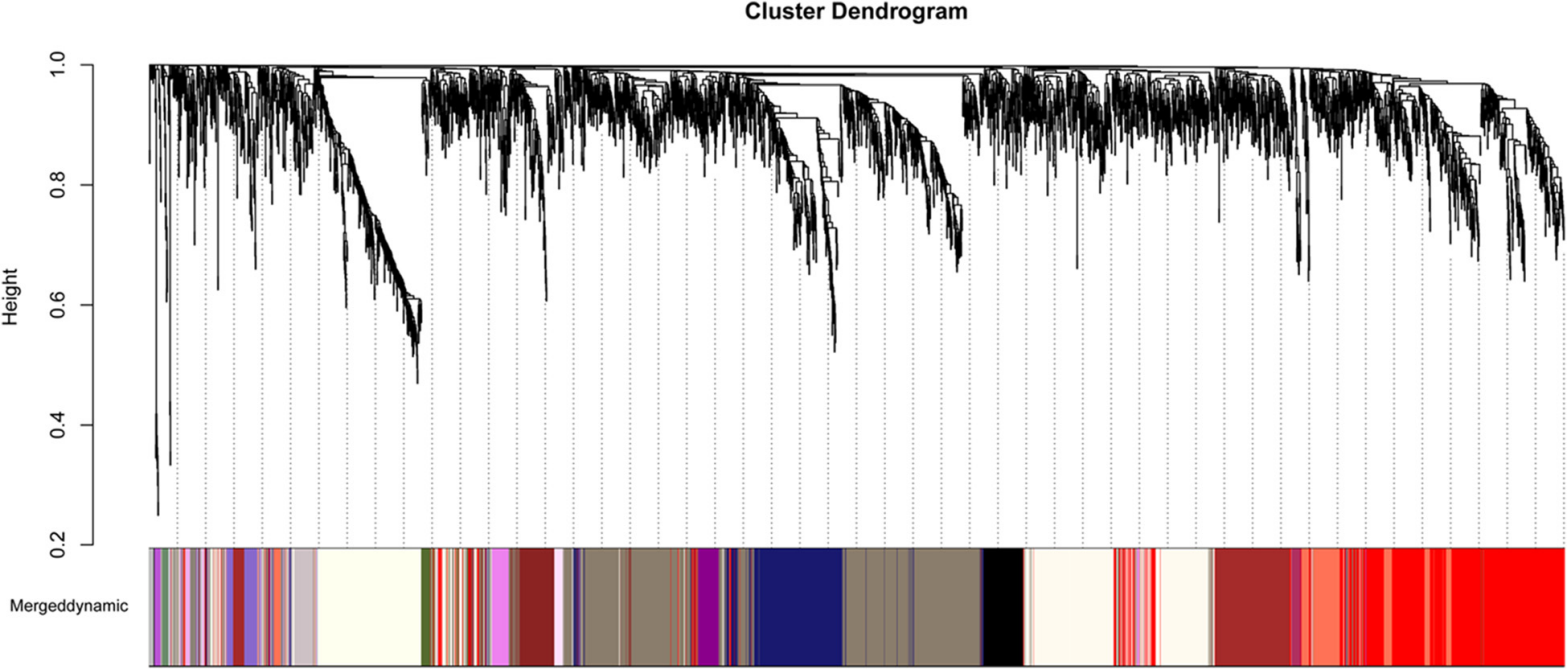
Identification of gene co-expression modules in CAD via hierarchical average linkage clustering (Dynamic tree cut algorithm was used to identify modules, and genes in the same branch could be assigned to different modules)

Using WGCNA package in R, the DEGs with similar patterns of expression were grouped into modules via hierarchical average linkage clustering. And a total of 21 modules (Fig. 1) were identified.

**Fig. 2:**
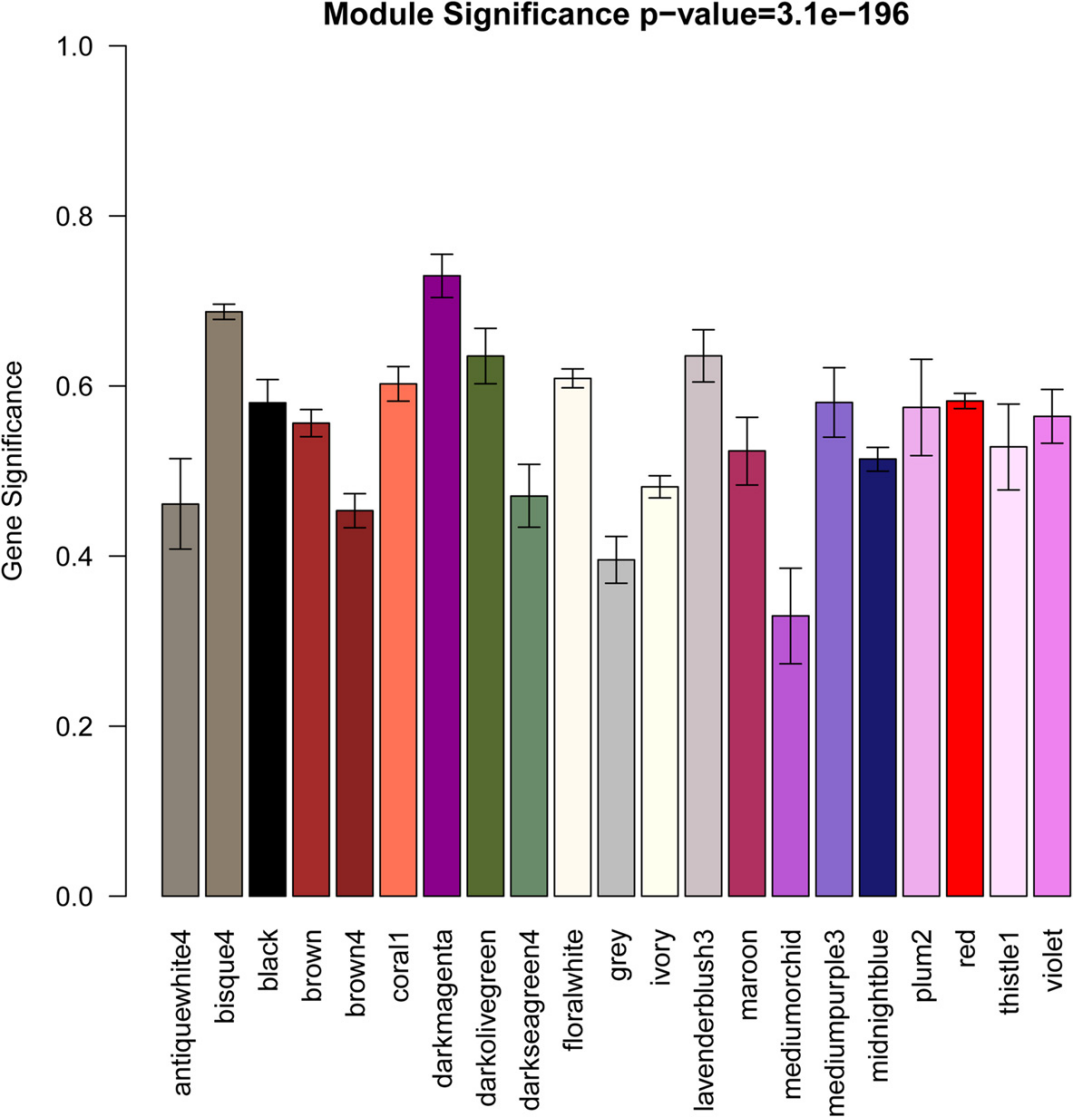
Module significance (MS) values of each module (Different colors of column indicated different modules)

Two methods were used to test the relevance between each module and the disease. Firstly, the MS value of each module was calculated, and modules with greater MS values were considered to have more connection with the disease (Fig. 2). We found that the darkmagenta module had the highest MS value among all of the selected modules. Afterwards, the relevance between each module and the disease were tested through calculating the relevance between the feature vectors of modules and phenotypes, and then all modules were ranked according to the significant *p*-value (Table 1). As could be seen in the table, the darkmagenta module was still the most relevant module. The results of the two methods were identical with each other. So the darkmagenta module was identified as the module highly relevant to CAD.

**Table 1:** Correlations between modules and coronary artery disease

Module	Correlation	*P-*value
Darkmagenta	0.88	1.95E-5
Lavenderblush3	0.76	1.07E-3
Coral1	0.75	1.20E-3
Grey	0.74	1.65E-3
Plum2	0.72	2.24E-3
Red	0.71	3.05E-3
Mediumpurple3	0.7	3.89E-3
Violet	0.68	5.44E-3
Thistle1	0.62	1.32E-2
Maroon	0.58	2.33E-2
Darkseagreen4	0.55	3.44E-2
Ivory	0.54	3.93E-2
Brown4	0.53	4.13E-2
Antiquewhite4	0.53	4.10E-2
Mediumorchid	−0.38	1.65E-1
Midnightblue	−0.6	1.75E-2
Brown	−0.69	4.45E-3
Black	−0.73	2.11E-3
Darkolivegreen	−0.74	1.66E-3
Floralwhite	−0.8	3.91E-4
Bisque4	−0.87	2.07E-5

### Functional and pathway enrichment analysis

Functional and pathway enrichment analysis were performed for the genes in the darkmagenta module. The significantly enriched functions mainly were membrane-associated biological processes and cellular components, and the enriched pathway included hypertrophic cardiomyopathy (HCM) (Table 2).

**Table 2:** GO and KEGG pathway enrichment analysis of darkmagenta module

Category	Term	Count	*P*-value
KEGG_PATHWAY	hsa05410:Hypertrophic cardiomyopathy (HCM)	3	2.85E-2
GOTERM_BP_FAT	GO:0016044~membrane organization	5	1.47E-2
GOTERM_BP_FAT	GO:0042391~regulation of membrane potential	3	4.44E-2
GOTERM_CC_FAT	GO:0005886~plasma membrane	18	4.37E-3
GOTERM_CC_FAT	GO:0044459~plasma membrane part	12	1.47E-2
GOTERM_CC_FAT	GO:0045121~membrane raft	3	4.95E-2

GOTERM_BP_FAT: Gene ontology term biological process; GOTERM_CC_FAT: Gene ontology term cellular component; KEGG: Kyoto Encyclopedia of Genes and Genomes

### Sub-pathway analysis of hub genes in darkmagenta module

**Fig. 3:**
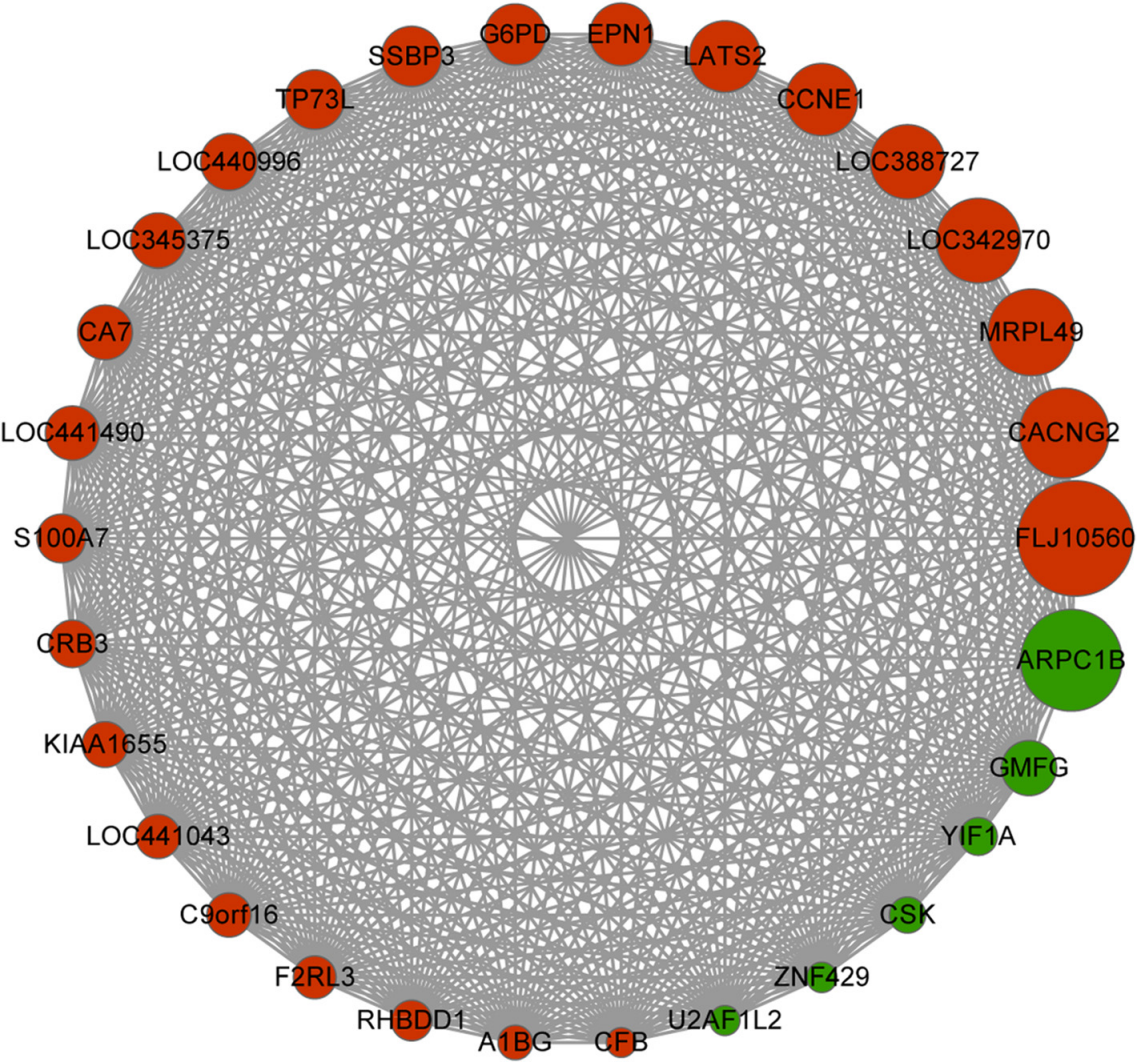
Network of top 30 genes in darkmagenta module (Node size:larger indicates a more significant differentially expressed gene, smaller indicates a less significant differentially expressed gene. Node color:Red indicates up-regulated gene, Green indicates down-regulated gene)

**Fig. 4:**
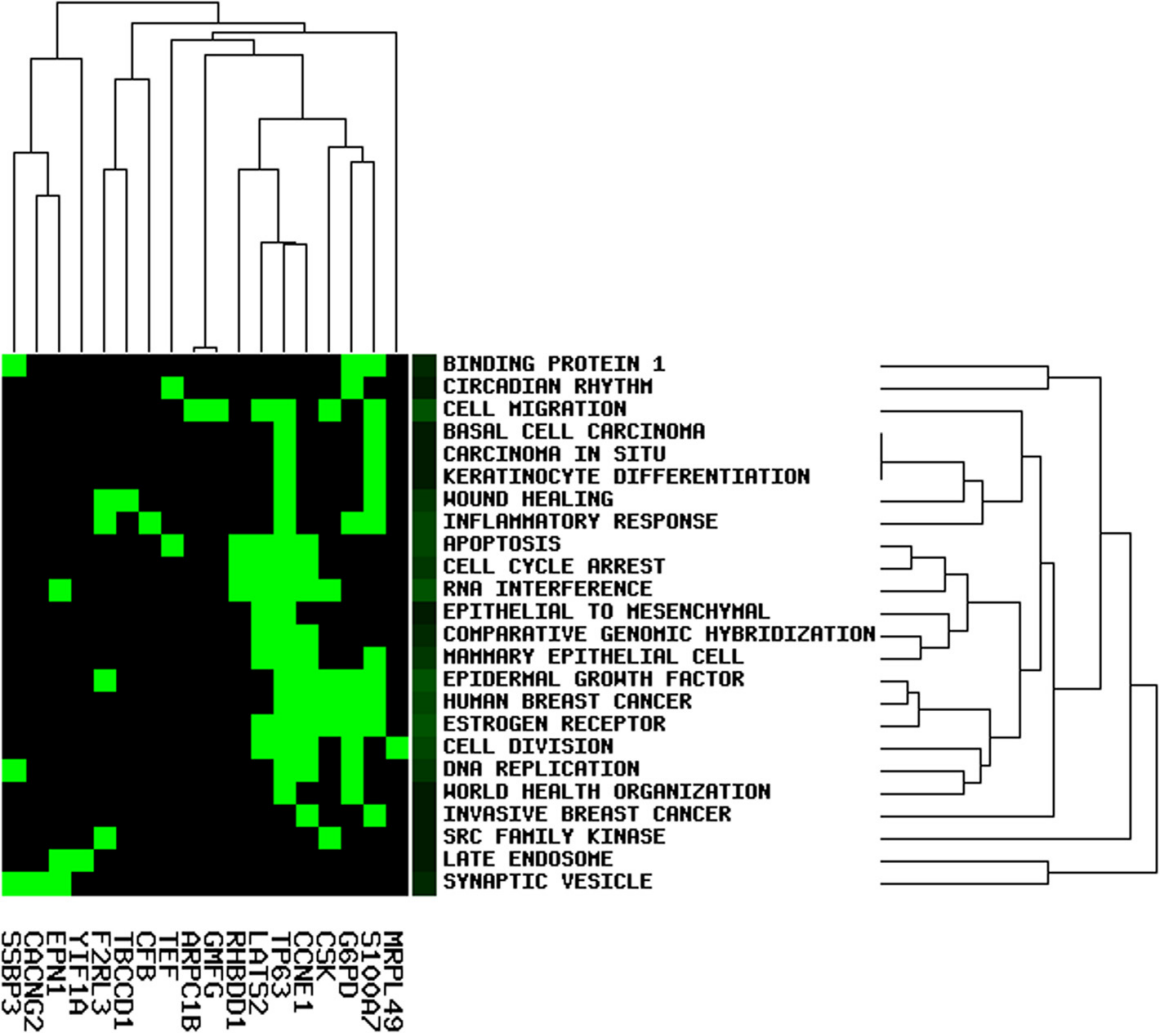
Enrichment analysis of functional items in biological processes for top 30 genes in darkmagenta module (Green indicates that the gene is enriched in the item, black indicates that the gene is not enriched in the item)

Highly connected hub genes in a module play important roles in biological processes. Therefore, the top 30 genes (Fig. 3) with the highest connectivity in darkmagenta module were taken as hub genes, including S100 calcium binding protein A7A (*S100A7*), tumor protein p63 (*TP63*), coagulation factor II (thrombin) receptor-like 3 (*F2RL3*), TBCC domain containing 1 (*TBCCD1*), glucose-6-phodphate dehydrogenase (*G6PD*) and carbonic anhydrase VII (*CA7*). Subsequently, iSubpathwayMiner package was used to identify sub-pathways of these hub genes (Table 3), and found two genes (*G6PD* and *CA7*) were enriched in the sub-pathways of pentose phosphate pathway and nitrogen metabolism. Data mining using GenCLiP 2.0 tools showed that 18 of the 30 hub genes were enriched in several functional items in biological process category, such as apoptosis, cell cycle arrest, epidermal growth factor, and wound healing (Fig 4).

**Table 3:** Sub-pathway analysis of top 30 genes in darkmagenta module

Pathway Id	Pathway name	*P*-value	Genes
path:00030_5	Pentose phosphate pathway	9.60E-3	G6PD
path:00030_4	Pentose phosphate pathway	2.31E-2	G6PD
path:00910_2	Nitrogen metabolism	3.12E-2	CA7
path:00030_3	Pentose phosphate pathway	3.65E-2	G6PD

## Discussion

In 2011, Grayson et al. perform microarray data analysis and find that genes implicated in activation of NF-kB were up-regulated in CAD [16]. Via network-driven integrative analysis, Huan et al. screen genes associated with coronary heart disease, and define network structure that shows the interactions of disease risk-related genes [30]. Using WGCNA, Tan et al. identify two modules closely related to atrial fibrillation in human left atrial tissues [31]. Here, the same data by Grayson et al. [16] were investigated by WGCNA to construct the co-expression network of CAD, which considered not only DEGs but also their interactions. Hierarchical average linkage clustering analysis was performed to group co-expressed genes into modules, and 21 modules were identified. The darkmagenta module was the most significant module identified by both MS and feature vector. Genes in darkmagenta module were mainly enriched in membrane-related functions, suggesting that genes in the darkmagenta module might play important roles in membrane functions during CAD. Pathway enrichment analysis indicated that genes in the darkmagenta module were enriched in hypertrophic cardiomyopathy (HCM) pathway. HCM is one of the most common inherited cardiac disorders, and previous studies demonstrate that CAD usually has adverse effects on the prognosis of patients with HCM [32, 33].

We listed the top 30 hub genes with the highest connectivity in the darkmagenta module, such as *S100A7*, *TP63*, *F2RL3*, *TBCCD1*, *G6PD* and *CA7*. Sub-pathway analysis showed that *G6PD* may might exert its role by influencing pentose phosphate pathway. Glucose-6-phosphate dehydrogenase (*G6PD*) is the rate-controlling enzyme of the pentose phosphate pathway [34], which is reported to be implicated in heart disease [35]. Results of spectrophotometry shows that the level of *G6PD* was significantly decreased in CAD patients [36]. The G6PD-deficient phenotype can protect against coronary heart disease by inhibiting 3-hydroxy-3-methylglutarylcoenzyme A reductase (HMG-CoA R) activity and reducing NADPH oxidase activity [37–39]. Previous study reports that G6PD can relax coronary artery by increasing Ca2+ sequestration, inhibiting Rho kinase and decreasing Ca2+ influx [40]. There were are also some studies revealed the multiple mechanisms of pentose phosphate pathway in bovine coronary arteries [41, 42]. Correlated fFunctions of involved the hub genes included apoptosis, cell cycle arrest, epidermal growth factor, and wound healing. *S100A7* was enriched in wound healing function. Following an injury, series of events that restore integrity and function toof a damaged tissue would occur in would healing process. For myocardial infarction, healing is essential for further prognosis [43]. *S100A7*, also called psoriasin, is a member of the S100 multigene family. S100 protein is proved to have play a role in wound healing, and *S100A7* is activated during wound healing [44]. Increased plasma levels of S100A8 and S100A9 can serve as marks for human cardiovascular disease, and their deletion protects aganist atherosclerosis to some degree [45]. As Another another number in S100 family, *S100A12* is reported to function as a prediction marker for cardiovascular events in chronic coronary artery diseaseCAD [46].

## Conclusions

In conclusion, total 3711 DEGs and 21 modules for them were identified in CAD samples. Via further analysis of the top 30 genes with highest connectivity in the most significant module, *G6PD* and *S100A7* were identified to be potential targets in CAD. However, the small sample size is a limitation of the study, and further studies are still needed to verify our findings.

## Highlights

1. A total of 21 significant modules were identified.
2. The most significant module was detected by module significance and feature vector.
3. G6PD in the module was predicted as candidate gene by enrichment analysis.
4. S100A7 involved in coronary artery disease by participating in wound healing.

## Abbreviations

CADm: coronary artery disease
DAVID: database for annotation, visualization, and integrated discovery
G6PD: glucose-6-phosphate dehydrogenase
GO: gene ontology
GS: gene significance
HCM: hypertrophic cardiomyopathy
MS: module significance
WGCNA: weighted gene co-expression network analysis

## References

[1] Ohira T, Iso H (2013). Cardiovascular disease epidemiology in Asia: an overview. Circ J.

[2] Hata J, Kiyohara Y (2013). Epidemiology of stroke and coronary artery disease in Asia. Circ J.

[3] Lozano R, Naghavi M, Foreman K, Lim S, Shibuya K, Aboyans V (2013). Global and regional mortality from 235 causes of death for 20 age groups in 1990 and 2010: a systematic analysis for the Global Burden of Disease Study 2010. Lancet.

[4] McCullough PA (2007). Coronary artery disease. Clin J Am Soc Nephrol.

[5] Simon AS, Vijayakumar T (2013). Molecular Studies on Coronary Artery Disease—A Review. Indian J Clin Biochem.

[6] Das S, Yadav D, Narang R, Das N (2002). Interrelationship between lipid peroxidation, ascorbic acid and superoxide dismutase in coronary artery disease. Curr Sci-Bangalore.

[7] Wild PS, Zeller T, Schillert A, Szymczak S, Sinning CR, Deiseroth A, et al. A genome-wide association study identifies LIPA as a susceptibility gene for coronary artery disease. Circ Cardiovasc Genet. 2011;4:403-12. 10.1161/CIRCGENETICS.110.958728 PMC3157552 21606135

[8] Kastrup J, Jørgensen E, Fuchs S, Nikol S, Bøtker HE, Gyöngyösi M (2011). A randomised, double-blind, placebo-controlled, multicentre study of the safety and efficacy of BIOBYPASS (AdGVVEGF121. 10NH) gene therapy in patients with refractory advanced coronary artery disease: the NOVA trial. EuroIntervention.

[9] Consortium I-RMRA (2012). The interleukin-6 receptor as a target for prevention of coronary heart disease: a mendelian randomisation analysis. Lancet.

[10] Chen F, Zhao X, Peng J, Bo L, Fan B, Ma D (2014). Integrated microRNA-mRNA analysis of coronary artery disease. Mol Biol Rep.

[11] Horvath S, Dong J (2008). Geometric interpretation of gene coexpression network analysis. PLoS Comput Biol.

[12] Ruan J, Dean AK, Zhang W (2010). A general co-expression network-based approach to gene expression analysis: comparison and applications. BMC Syst Biol.

[13] Malki K, Tosto MG, Jumabhoy I, Lourdusamy A, Sluyter F, Craig I (2013). Integrative mouse and human mRNA studies using WGCNA nominates novel candidate genes involved in the pathogenesis of major depressive disorder. Pharmacogenomics.

[14] Udyavar AR, Hoeksema MD, Clark JE, Zou Y, Tang Z, Li Z (2013). Co-expression network analysis identifies Spleen Tyrosine Kinase (SYK) as a candidate oncogenic driver in a subset of small-cell lung cancer. BMC Sys Biol.

[15] Zhao H, Cai W, Su S, Zhi D, Lu J, Liu S. Screening genes crucial for pediatric pilocytic astrocytoma using weighted gene coexpression network analysis combined with methylation data analysis. Cancer Gene Ther. 2014. 10.1038/cgt.2014.49 25257306

[16] Grayson BL, Wang L, Aune TM (2011). Peripheral blood gene expression profiles in metabolic syndrome, coronary artery disease and type 2 diabetes. Genes Immun.

[17] Barrett T, Troup DB, Wilhite SE, Ledoux P, Rudnev D, Evangelista C (2007). NCBI GEO: mining tens of millions of expression profiles--database and tools update. Nucleic Acids Res.

[18] Edgar R, Domrachev M, Lash AE (2002). Gene Expression Omnibus: NCBI gene expression and hybridization array data repository. Nucleic Acids Res.

[19] Bolstad BM, Irizarry RA, Astrand M, Speed TP. A comparison of normalization methods for high density oligonucleotide array data based on variance and bias.Bioinformatics. 2003;19:185-93. 10.1093/bioinformatics/19.2.185 12538238

[20] Smyth GK. Linear models and empirical bayes methods for assessing differential expression in microarray experiments. Stat Appl Genet Mol Biol. 2004;3 (Article 3). 10.2202/1544-6115.1027 16646809

[21] Yip AM, Horvath S (2007). Gene network interconnectedness and the generalized topological overlap measure. BMC Bioinforma.

[22] Ravasz E, Somera AL, Mongru DA, Oltvai ZN, Barabasi AL (2002). Hierarchical organization of modularity in metabolic networks. Science.

[23] Langfelder P, Horvath S (2008). WGCNA: an R package for weighted correlation network analysis. BMC Bioinforma.

[24] Mason MJ, Fan G, Plath K, Zhou Q, Horvath S (2009). Signed weighted gene co-expression network analysis of transcriptional regulation in murine embryonic stem cells. BMC Genomics.

[25] Tweedie S, Ashburner M, Falls K, Leyland P, McQuilton P, Marygold S (2009). FlyBase: enhancing Drosophila gene ontology annotations. Nucl Acids Res.

[26] Kanehisa M, Goto S (2000). KEGG: kyoto encyclopedia of genes and genomes. Nucl Acids Res.

[27] Huang DW, Sherman BT, Tan Q, Kir J, Liu D, Bryant D (2007). DAVID Bioinformatics Resources: expanded annotation database and novel algorithms to better extract biology from large gene lists. Nucl Acids Res.

[28] Li C, Li X, Miao Y, Wang Q, Jiang W, et al. SubpathwayMiner: a software package for flexible identification of pathways.Nucleic Acids Res.2009;37:e131-e131 10.1093/nar/gkp667 PMC2770656 19706733

[29] Wang JH, Zhao LF, Lin P, Su XR, Chen SJ, Huang LQ, et al. GenCLiP 2.0: a web server for functional clustering of genes and construction of molecular networks based on free terms.Bioinformatics. 2014;30:2534-6. 10.1093/bioinformatics/btu241 24764463

[30] Huan T, Zhang B, Wang Z, Joehanes R, Zhu J, Johnson AD (2013). A systems biology framework identifies molecular underpinnings of coronary heart disease. Arterioscler Thromb Vasc Biol.

[31] Tan N, Chung MK, Smith JD, Hsu J, Serre D, Newton DW, et al. A Weighted Gene Co-Expression Network Analysis of Human Left Atrial Tissue Identifies Gene Modules Associated withAtrial Fibrillation. Circ Cardiovasc Genet. 2013;6:362-71. 10.1161/CIRCGENETICS.113.000133 PMC3955718 23863953

[32] Sorajja P, Ommen SR, Nishimura RA, Gersh BJ, Berger PB, Tajik AJ (2003). Adverse prognosis of patients with hypertrophic cardiomyopathy who have epicardial coronary artery disease. Circulation.

[33] Okayama S, Soeda T, Kawakami R, Takami Y, Somekawa S, Ueda T, et al. Evaluation of coronary artery disease and cardiac morphology and function in patients with hypertrophic cardiomyopathy, using cardiac computed tomography. Heart Vessel. 2013;1–8. 10.1007/s00380-013-0452-9 24326884

[34] Stover NA, Dixon TA, Cavalcanti AR (2011). Multiple independent fusions of glucose-6-phosphate dehydrogenase with enzymes in the pentose phosphate pathway. PLoS One.

[35] Brewer AC, Mustafi SB, Murray TV, Rajasekaran NS, Benjamin IJ (2013). Reductive stress linked to small HSPs, G6PD, and Nrf2 pathways in heart disease. Antioxid Redox Signal.

[36] Serdar Z, Aslan K, Dirican M, Sarandöl E, Yeşilbursa D, Serdar A (2006). Lipid and protein oxidation and antioxidant status in patients with angiographically proven coronary artery disease. Clin Biochem.

[37] Meloni L, Manca M, Loddo I, Cioglia G, Cocco P, Schwartz A (2008). Glucose-6-phosphate dehydrogenase deficiency protects against coronary heart disease. J Inherit Metab Dis.

[38] Matsui R, Xu S, Maitland KA, Hayes A, Leopold JA, Handy DE (2005). Glucose-6 phosphate dehydrogenase deficiency decreases the vascular response to angiotensin II. Circulation.

[39] Hecker PA, Leopold JA, Gupte SA, Recchia FA, Stanley WC (2013). Impact of glucose-6-phosphate dehydrogenase deficiency on the pathophysiology of cardiovascular disease. Am J Physiol Heart Circ Physiol.

[40] Ata H, Rawat DK, Lincoln T, Gupte SA (2011). Mechanism of glucose-6-phosphate dehydrogenase-mediated regulation of coronary artery contractility. Am J Physiol Heart Circ Physiol.

[41] Gupte SA, Arshad M, Viola S, Kaminski PM, Ungvari Z, Rabbani G (2003). Pentose phosphate pathway coordinates multiple redox-controlled relaxing mechanisms in bovine coronary arteries. Am J Physiol Heart Circ Physiol.

[42] Larsen BT, Gutterman DD (2006). Hypoxia, coronary dilation, and the pentose phosphate pathway. Am J Physiol Heart Circ Physiol.

[43] Frantz S, Bauersachs J, Ertl G. Post-infarct remodelling: contribution of wound healing and inflammation. Cardiovasc Res. 2008. 10.1093/cvr/cvn292 PMC2639128 18977766

[44] Lee KC, Eckert RL (2006). S100A7 (Psoriasin)–mechanism of antibacterial action in wounds. J Investig Dermatol.

[45] Averill MM, Kerkhoff C, Bornfeldt KE (2012). S100A8 and S100A9 in cardiovascular biology and disease. Arterioscler Thromb Vasc Biol.

[46] Saito T, Hojo Y (2011). Ogoyama: S100A12 as a marker to predict cardiovascular events in patients with chronic coronary artery disease. Circ J.

